# Twelve eyes see more than eight. Referee bias and the introduction of additional assistant referees in soccer

**DOI:** 10.1371/journal.pone.0227758

**Published:** 2020-02-26

**Authors:** Andrea Albanese, Stijn Baert, Olivier Verstraeten

**Affiliations:** 1 Luxembourg Institute of Socio-Economic Research, Esch-sur-Alzette/Belval, Luxembourg; 2 Ghent University, Ghent, Belgium; 3 IZA, Bonn, Germany; 4 GLO, Maastricht, Netherlands; 5 Research Foundation–Flanders, Brussels, Belgium; 6 University of Antwerp, Antwerp, Belgium; 7 Université Catholique de Louvain, Louvain-la-neuve, Belgium; 8 IMISCOE, Rotterdam, Netherlands; Queen Mary University of London, UNITED KINGDOM

## Abstract

This study is the first to investigate whether the introduction of additional assistant referees in the UEFA Europa League (2009–2010 season) and the UEFA Champions League (2010–2011 season) was associated with lower referee bias in terms of home and “big” team favouritism. To this end, we analyse a unique database with pre- and within-game characteristics of all games in seven recent seasons in these leagues by means of bivariate probit regression models. We find evidence for substantial referee bias before the introduction of additional referees, while no such evidence is found after the introduction. Furthermore, additional assistants go hand in hand with more yellow cards for both home and away teams. We show that these findings are robust to multiple operationalisations of referee bias and that they are not just picking up a general time evolution towards less referee bias or the effect of parallel reforms.

## I. Introduction

Soccer referees are often accused of partial decision-making. For example, after the 2016–2017 season quarter-final return of the Union of European Football Association (UEFA) Champions League between Real Madrid and Bayern Munich, the referee was criticised for giving an unjustified second yellow card to Bayern Munich player Vidal and not giving a second yellow card to Real Madrid player Casemiro [1]. Moreover, Real Madrid player Ronaldo scored in extra time from an offside position. Other famous controversial cases are the 2016–2017 season quarter-final return of the UEFA Champions League between FC Barcelona and Paris Saint-Germain [2] and the 2012–2013 season quarter-final return of the UEFA Champions League between Dortmund and Malaga [3]. These criticisms suggest that the referee’s decisions were biased.

Referee bias may be defined as systematic decisions in favour of certain teams such as home or “big” teams, which are considered to be unfair (e.g. [4]). Such a preferential treatment, which can result from conscious bias as well as from human error or incompetence, may have a big economic impact on the teams. For example, Real Madrid eventually won the tournament and received 26 million euros more in prize money than Bayern Munich [5]. Similarly, by the end of the respective tournaments, FC Barcelona and Borussia Dortmund ended up winning 5 and 22 million euros more in prize and television money than Paris Saint-Germain and Malaga [5, 6]. However, it does not end there. Other revenues, such as ticket sales and merchandise, can be affected by team performance as well (e.g. [7]).

Therefore, not surprisingly, many academics have investigated the popular idea of referee bias in European soccer. As reviewed by [4], referee bias is not just a myth and it affects different aspects of the game such as allowance for time lost (also known as the ±1 bias), foul calls, and the awarding of yellow and red cards (e.g. [8, 9, 10]). The fast pace of soccer and the need for quick decisions can sometimes lead the referee to human error (e.g. [11, 12]). While errors can randomly affect both teams in the game, certain factors can systematically favour certain teams. Previous literature has shown that the presence of big crowds or of important teams in a game may affect the decisions of referees, who, despite an intrinsic motivation for impartiality, may unconsciously avoid social sanctioning by the crowd or the media (e.g. [13, 14]).

It can be argued that referee bias may be attenuated by improving the referee control of the game and reducing the number of ‘uncertain’ calls that he has to take during the match. Indeed, a referee can be more easily influenced by the crowd or the media when he is uncertain about the correct decision to take. As argued by a former UEFA chief refereeing officer, Pierluigi Collina, a better control of the game might be obtained by adding additional assistant referees, partly because they allow the other four (assistant) referees to focus on their core tasks [12]. Our hypothesis is therefore that introducing additional assistants may, in turn, decrease the likelihood of biased decisions during a match.

Before the 2009–2010 season, there were four referees at every game in the UEFA Champions League and the UEFA Europa League: the main referee and three assistants. Two assistant referees, also known as linesmen, assisted the main referee particularly with offside situations and out of play ball decisions. Furthermore, a fourth official assisted the referee with various off-field matters such as managing substitutions and overseeing the technical area. In the 2009–2010 season, UEFA introduced two additional assistant referees as a trial during all games played in the UEFA Europa League. In the following season, this approach was extended to all games played in the UEFA Champions League. The two additional referees work behind the goal line and assist the referee in deciding situations in or near the penalty area. The additional assistant referees are now ubiquitous in European international club soccer [15].

In this paper we focus on referee bias in terms of systematic preferential treatment for home and “big” teams. Following a strand of the literature on referee bias (e.g. [8, 9, 10]), we focus on the number of yellow cards awarded to the home and away teams due to the discretionary power that referees have in awarding yellow cards for fouls and misconduct. Our contribution to the literature is twofold. First, we provide more recent evidence on favouritism for home and “big” teams in the two main European competitions: the UEFA Champions League and UEFA Europa League between 2007 and 2014. As in the previous literature (e.g. [16, 17]), we also test whether a running track separating the supporters from the pitch reduces favouritism. Second, we test whether home and “big” team favouritism changed after the introduction of additional referees. To the best of our knowledge, our study is the first to investigate whether additional assistant referees can reduce this type of referee bias.

## II. Literature on referee bias

The previous literature has analysed several determinants of referee bias such as social pressure from the crowd and the media (e.g. [18]), cultural closeness of the referee to the team [19, 20], players’ height [21], uniform colour [22], and ethnicity [23]. In this section, we provide some examples of papers analysing favouritism for home and "big" teams and the factors that may influence it. For a systematic literature review on soccer refereeing and referee bias, we refer to [24].

### Home Favouritism

A large proportion of the studies have focused on home team favouritism. For instance, [16, 17, 25–28] showed, based on data from Spanish, German, Italian, English, and Brazilian national competitions, that the injury time awarded by the referee in the second half of a soccer game is significantly longer if the away team is one goal ahead compared with when the home team is leading by one goal. Furthermore, [16, 29, 30] showed that in the German and English championships, home teams tend to be awarded more penalty kicks in their favour. Finally, home favouritism also exists in the awarding of yellow and red cards in the English, Italian, German, Spanish, and UEFA competitions [8, 9, 31, 32], although [33] and [34] could not replicate the significant results in the English League. Besides, by analysing a large sample of English matches, [35] found that home favouritism may even affect the final score of a game.

### “Big” Team Favouritism

Besides home favouritism, referees may be biased by the importance of the teams. [36] found that over the 2014–2015 Spanish Liga season, referees allowed more time for more popular teams when they were behind and less time when they were ahead, potentially due to the referees’ internalisation of the preferences of the media and supporters. Similarly, [10] showed that teams from the big five European competitions (English Premier League, Spanish Primera Division, French Ligue 1, German Bundesliga, and Italian Serie A) receive fewer yellow cards when playing against a team outside of the big five in European international club soccer, keeping team strength constant. Finally, [37] showed that in the 2011–2012 English Premier League, home favouritism in terms of penalty kicks occurred only for the top two teams (Manchester City and Manchester United).

### Factors Influencing Home and “Big” Team Favouritism

Preferential treatment for home and “big” teams appears to be heterogeneous in terms of match characteristics. First, [25] found that in the Spanish Primera Division, the magnitude of home team bias in terms of injury time was affected by the stakes: it increased when the point system for winning the game increased from 2 to 3 points and was higher towards the end of the season. Second, other papers have found that a running track separating the pitch from the crowd can reduce home favouritism [8, 9, 10, 16, 17]. As previous literature has shown that physical distance is an important driver of the intensity of social interaction (e.g. [38]), attenuated home favouritism in the presence of a running track might suggest that crowds that are physically closer to referees can exercise social pressure more effectively. Third, crowd size was found to increase extra-time in favour of the home team in Spain [25]. Similarly, it also increases home favouritism for yellow and red cards in England [30, 39], Italy [32], and Spain [9]. In contrast, in the 2009–2010 and 2010–2011 UEFA Champions and Europa League, it was crowd density that drove home favouritism [40].

## III. Methods

### III.1 Data

To answer our research question, we constructed a dataset containing all games played in the UEFA Champions League and UEFA Europa League between the 2007–2008 and 2013–2014 seasons, except for final games (for which no home and away games are designated). After the preliminaries, which begin with 78 teams, the UEFA Champions League starts with a group stage of 32 teams divided into groups of four teams. The first- and second-place teams qualify for the knock-out stage of the UEFA Champions League, and the third-place teams proceed to the knock-out stage of the UEFA Europa League. Since the 2009–2010 season, the UEFA Europa League has started the season with a group stage of 48 teams divided into groups of four teams. The winners and runners-up of each group go through to the knock-out stage. During the 2007–2008 and 2008–2009 seasons, the format of the UEFA Europa League, then called the ‘UEFA Cup’, was different. The group stage starts with 40 teams divided into groups of five teams. In contrast to the current format, there was only one encounter between the teams, with each team playing twice at home and twice away. The three first-place teams in each group qualified for the knock-out stage. In the knock-out stage of both competitions, two teams played against each other both at home and away, and the team that scored the most goals over those two games proceeded to the next round.

In total, our dataset contains 2,168 games, of which 868 are from the UEFA Champions League and 1,300 are from the Europa League. These games were played between 189 different soccer teams. Unlike most studies on referee bias (e.g. [16, 17]), we do not use the game as the unit of observation. Instead, we analyse our data at the minute level, in line with [8] and [9]. This approach results in a dataset of 195,120 observations at the game-minute level. We explain below how we take into account that (i) these observations are clustered at the game level and (ii) minute 45 and minute 90 may take more than one minute due to injury time.

We follow a strand of the literature (e.g. [8, 10]) and measure home and “big” team favouritism as the number of yellow cards awarded in two different analyses. In a first analysis, we test the presence of home favouritism, which can result in a lower (higher) probability of the home (away) team receiving a yellow card. In line with previous literature, we expect that home teams will also be less favoured if there is a track separating the crowd from the field. Without such a track, the referee will be more intimidated by the home crowd. As explained, our hypothesis is that these track effects should also be lower in games where a fifth and a sixth referee are present.

In a second analysis, we follow [10] and test whether teams from the big five European soccer competitions were favoured in the awarding of yellow cards as a result of the prestige of these competitions. More specifically, our hypothesis is that the big five teams have a lower chance of receiving a yellow card in a given minute when playing against non-big-five teams, keeping the teams’ relative strength constant. Again, we expect that this favouritism will be lower in games where additional assistant referees are present.

To test these hypotheses, numerous variables were collected with respect to the included games. The first and most important source was the game reports available on the official UEFA website (http://www.uefa.com). Since the crowd size during these games and the names of the referees were not always available in these reports, we augmented the data from these reports with additional information from Worldfootball (http://www.worldfootball.net). Table 1 presents the descriptive statistics for the variables used in our statistical analyses.

**Table 1:** Data: Summary statistics.

	Mean	SD
**A. Dependent variables**		
Yellow card–home	0.020	-
Yellow card–away	0.025	-
**B. Independent variables**		
Track	0.174	-
Home big five	0.454	-
Away big five	0.454	-
Additional assistant referees	0.699	-
**C. Control variables**		
*C1*. *Pre-game information*		
Attendance	30,083.220	19,430.880
Relative strength	5.158	14.863
Derby	0.017	-
*C2*. *Within-game information*		
Goal difference	0.200	1.164
Yellow card three minutes prior–home	0.052	-
Yellow card three minutes prior–away	0.066	-
Number of prior yellow cards–home	0.644	-
Number of prior yellow cards–away	0.839	-
Prior red card–home	0.023	-
Prior red card–away	0.030	-
N	195,120	

A definition of these variables can be found in Section III.1. No standard deviation (SD) is provided for binary variables.

We used a pair of dependent variables, i.e. the awarding of a yellow card to the home team and to the away team in a given minute. Both are dummy variables and thus take the value 1 if a yellow card has been awarded to the team under review and 0 otherwise. As can be seen in Panel A of Table 1, a yellow card is awarded to a home team in approximately one minute out of fifty, while an away team receives a yellow card in one minute out of forty. We investigated whether this home advantage in receiving yellow cards is related to referee bias (versus whether it is fully explained by other reasons for a home advantage; [30, 33, 41]).

Our first independent variable is a binary variable that captures the presence of a running track between the field and the spectators. In total, 377 of the 2,168 analysed games were played in a stadium with a running track. The second and third independent variables indicate whether the home and away teams come from one of the big five European soccer competitions: the English Premier League, Spanish Primera Division, Italian Serie A, German Bundesliga, and the French Ligue 1 [10]. Given that all teams play as many games at home as they do away, the mean value for both variables is the same (and equal to 0.454). The fourth and final independent variable captures the presence of a fifth and sixth referee. These additional assistant referees were present in 69.9 percent of the analysed games.

Finally, Panel C of Table 1 shows various control variables. We included these variables since they are possible confounders of the association between our independent variables and outcome. First, according to the theory they are likely to influence the chance of being awarded a yellow card. Moreover, they are also correlated with the independent variables. We check this by implementing three separate probit models using as an outcome the independent variables presence of a running track, teams from the big five competitions and additional assistants. The Wald test always reject the null for the joint significance with a *p-value* of 0.000 and a Pseudo R^2^ ranging between 0.13 and 0.03. Therefore, their inclusion in the main model should not only increase efficiency but also reduce bias (e.g. [42]). These control variables can be divided into pre-game and within-game influences.

The first pre-game control variable captures crowd size, which can affect the award of yellow cards for home and away teams in different away. Indeed, the presence of large crowd may intimidate the referee and deter (encourage) the awards of yellow cards towards the home (away) team, which has been confirmed in the previous empirical literature (e.g. [16]). On average, approximately 30,083 individuals are in the stadium for UEFA Champions League and UEFA Europa League games. In our analyses, we included the logarithm of attendance. A second pre-game determinant of receiving yellow cards is the teams’ relative strength. Indeed, stronger teams may be able to incur in fewer sanctioning when facing a weaker team (e.g. [10]). In this respect, we followed [43] definition to create our proxy of relative strength based on the competing teams’ UEFA team coefficient. This coefficient is based on a team’s performance over the past five seasons of the UEFA Champions League and UEFA Europa League. More concretely, it is the quotient of the UEFA team coefficient of the home team divided by the coefficient of the away team for that season plus 1. A third pre-game control variable is an indicator of derby games. Indeed, a game between two clubs located close to each other will be a more intense encounter due to a stronger rivalry between players and supporters (e.g. [8]). Consequently, we needed to control for the higher probability of a player receiving a yellow card in a derby. Because all games in our dataset are international, like [41], we define a derby as a game between two teams from the same country.

Turning now to the within-game influences, an obvious one is the goal difference at the start of the analysed minute, calculated by subtracting the number of goals scored by the away team from the number of goals scored by the home team. This goal difference is expected to be negatively (positively) correlated with aggression by the home (away) team and, therefore, with that team being awarded yellow cards [31]. Other within-game influences involve previously awarded yellow and red cards. On the one hand, a yellow or red card given to a team can reduce the chances of another yellow or red card being given to the same team—the deterrence effect. On the other hand, referees tend to compensate, with the consequence that a yellow or red card given to one team may increase the chances of a yellow or red card being awarded to its opponent [8, 9]. Specifically, we included the following variables to capture these effects: (i) yellow card received by the home (away) team during the preceding three minutes of the game, (ii) number of yellow cards awarded to the home (away) team during the game, and (iii) red card received by the home (away) team during the game.

We included several within-game influences not presented in Table 1. These all control for the moment in the game. First, since the competitive spirit during the game may increase and affect the likelihood of a sanction, we included ‘minute’ and ‘minute squared’ as in [8, 9]. Second, ‘minute 45’ and ‘minute 90’ are incorporated as well. The objective of these dummies is to capture the events that happened during injury time at both half-time and at the end of the game. We did not include these variables in Table 1 because their descriptive statistics are the same for every game.

The data are available as S1 Dataset.

## III.2 Regression model

The data presented in Subsection III.1 were analysed by means of a bivariate probit model. Previous literature on referees bias has relied on data at the match-level (e.g. [10, 30, 31]). Instead, we follow some recent literature and use data at the minute-level (e.g. [8, 9, 44]). The main advantage of this approach is that we can take the flow of events that occur during the game into account. In the end, two games can have the same number of goals and yellow and red cards, but the order of events is completely different. For example, the advantage of a particular team during the game may have caused the opponent to play more aggressively and receive a red card, whereas in another game, a team may have received a red card that resulted in the other team scoring more easily. Finally, as the awarding of sanctions to one team cannot be assumed to be uncorrelated to the awarding of sanctions to the other team, we jointly estimated the probability of a yellow card being awarded to either the home or the away team (e.g. [8, 10, 31]). The bivariate probit model can be formalised by the following two-equation model:

$$y_{1t}^{*}=x_{1t}^{\prime }\beta_{1}+\varepsilon_{1t},$$

$$y_{2t}^{*}=x_{2t}^{\prime }\beta_{2}+\varepsilon_{2t}.$$ (1)

The observed binary outcomes of a yellow card to the home team (*y*_1*t*_) and to the away team (*y*_2*t*_) during a minute *t* are equal to 1 if the respective unobserved latent variables ($y_{1t}^{*}$ or $y_{2t}^{*}$) are larger than 0; otherwise, they take the value of zero. Two vectors of explanatory variables, $x_{1t}^{\prime }$ and $x_{2t}^{\prime },$ were included, which also contain our dummy variables of interest: the presence of additional assistant referees or of a track separating the pitch (see Subsection III.1). The error terms *ε*_1*t*_ and *ε*_2*t*_ are assumed to have a standard bivariate normal distribution with zero mean, unit variance, and correlation *ρ*. For a full derivation of the log likelihood function in a bivariate probit model, we refer the reader to [45], pages 710–712. In all models, we cluster the standard errors at the game level since the probabilities of receiving a yellow card for each minute are likely correlated within the same game.

## IV. Results

### IV.1 Home favouritism analysis

Table 2 presents the results of our analysis on home favouritism. In Model (1), yellow cards for the home and away teams are regressed on the control variables and one independent variable, i.e. the track variable. As noted, we expect that home teams are less favoured by referees in terms of the awarding of yellow cards when a track separates the crowd from the field. Indeed, the sign of the track variable is the expected sign, i.e. positive for home teams and negative for away teams. However, in contrast to [8, 9, 31], this variable is not significantly statistically different from 0. Thus, the evidence for referee bias in the sense that home (away) teams are awarded more (fewer) yellow cards when a track separates the crowd from the pitch is not replicated based on our more recent data.

**Table 2:** Regression results: Home-favouritism.

	(1)	(2)
**Dependent variable: Yellow card**–**home**		
Additional assistant referees × Track		-0.059 (0.039)
Additional assistant referees		0.074*** (0.017)
Track	0.028 (0.018)	0.072** (0.033)
Log attendance	-0.022** (0.009)	-0.016* (0.009)
Relative strength	-0.002* (0.001)	-0.002* (0.001)
Relative strength squared	0.000 (0.000)	0.000 (0.000)
Derby	-0.010 (0.044)	-0.006 (0.043)
Minute	0.016*** (0.001)	0.016*** (0.001)
Minute squared	-0.000*** (0.000)	-0.000*** (0.000)
Minute 45	0.475*** (0.045)	0.475*** (0.045)
Minute 90	0.933*** (0.038)	0.934*** (0.038)
Goal difference	-0.025*** (0.006)	-0.025*** (0.006)
Goal difference squared	-0.010*** (0.003)	-0.010*** (0.003)
Yellow card three minutes prior–home	-0.028 (0.031)	-0.028 (0.031)
Yellow card three minutes prior–away	0.023 (0.026)	0.023 (0.026)
Number of prior yellow cards–home	-0.016** (0.008)	-0.018** (0.008)
Number of prior yellow cards–away	0.023*** (0.008)	0.023*** (0.008)
Prior red card–home	0.045 (0.037)	0.042 (0.036)
Prior red card–away	0.021 (0.035)	0.017 (0.034)
Intercept	-2.370*** (0.091)	-2.477*** (0.095)
**Dependent variable: Yellow card away**		
Additional assistant referees × Track		0.071** (0.036)
Additional assistant referees		0.028* (0.015)
Track	-0.012 (0.016)	-0.063** (0.030)
Log attendance	0.030*** (0.009)	0.034*** (0.009)
Relative strength	0.002* (0.001)	0.002 (0.001)
Relative strength squared	0.000 (0.000)	-0.000 (0.000)
Derby	0.044 (0.049)	0.049 (0.048)
Minute	0.020*** (0.001)	0.020*** (0.001)
Minute squared	-0.000*** (0.000)	-0.000*** (0.000)
Minute 45	0.493*** (0.042)	0.493*** (0.042)
Minute 90	0.962*** (0.036)	0.962*** (0.036)
Goal difference	0.009 (0.006)	0.009 (0.006)
Goal difference squared	-0.018*** (0.003)	-0.019*** (0.003)
Yellow card three minutes prior–home	0.098*** (0.025)	0.098*** (0.025)
Yellow card three minutes prior–away	-0.025 (0.026)	-0.025 (0.026)
Number of prior yellow cards–home	0.023*** (0.008)	0.023*** (0.008)
Number of prior yellow cards–away	-0.040*** (0.007)	-0.041*** (0.007)
Prior red card–home	0.100*** (0.037)	0.096*** (0.037)
Prior red card–away	0.052 (0.035)	0.048 (0.035)
Intercept	-2.849*** (0.092)	-2.912*** (0.096)
Log pseudo-likelihood	-39,752.151	-39,736.903
N	195,120	

The presented statistics are bivariate probit model estimates and standard errors, clustered at the game level, in parentheses. A definition of the variables used can be found in Section III.1.

*** (**) ((*)) indicates significance at the 1% (5%) ((10%)) significance level.

In Model (2), we add two variables to Model (1): the variable capturing the presence of additional referees and the interaction between the latter variable and the track variable. Model (2) confirms our expectation of less referee bias after the adoption of a fifth and sixth referee. First, after the inclusion of the additional variables, the track variable becomes statistically significant with respect to both dependent variables. Thus, in the reference situation in which the additional assistant referees are not present, the home team has a statistically significant higher probability of receiving a yellow card (*b = 0*.*072*; *p = 0*.*032*) and the away team has a statistically significant lower probability of receiving a yellow card (*b = −0*.*063*; *p = 0*.*038*) in each minute if there is a track.

Second, this pattern disappears when additional referees are added. That is, the sum of the track variable and the interaction between this variable and the additional referees variable is economically and statistically insignificant with respect to the awarding of a yellow card, both for home teams (*b = 0*.*013 = 0*.*072 − 0*.*059*; *p = 0*.*549*) and away teams (*b = 0*.*008 = −0*.*063 + 0*.*071; p = 0*.*663*).

Third, the interaction between the track variable and the additional referees variable is statistically significant with respect to yellow cards for the away team (*b = 0*.*071*; *p = 0*.*046*) and is close to weak statistical significance with respect to yellow cards for the home team (*b = −0*.*059*; *p = 0*.*129*).

Fourth, the additional referees variable is highly significantly associated with yellow cards for the home team (*b = 0*.*074*; *p = 0*.*000*) and weakly significantly associated with yellow cards for the away team (*b = 0*.*028*; *p = 0*.*062*). Thus, after the introduction of a fifth and sixth referee, more yellow cards are awarded both to home and away teams.

### IV.2 “Big” team favouritism

Table 3 presents the results of our analysis on “big” team favouritism. Here, we inspected referee bias in terms of the favourable treatment of teams from the big five European soccer competitions. Again, we tested whether this operationalisation of referee bias is affected by the adoption of additional assistant referees.

**Table 3:** Regression results: Big five favouritism.

	(1)	(2)
**Dependent variable: Yellow card home**		
Additional assistant referees × Home big five		0.021 (0.031)
Additional assistant referees × Away big five		-0.047 (0.031)
Additional assistant referees		0.081*** (0.028)
Home big five	-0.022 (0.015)	-0.031 (0.027)
Away big five	0.031** (0.014)	0.070*** (0.027)
Track	0.023 (0.018)	0.023 (0.018)
Log attendance	-0.021** (0.010)	-0.017* (0.010)
Relative strength	-0.002* (0.001)	-0.002* (0.001)
Relative strength squared	0.000 (0.000)	0.000 (0.000)
Derby	-0.014 (0.044)	-0.010 (0.043)
Minute	0.016*** (0.001)	0.016*** (0.001)
Minute squared	-0.000*** (0.000)	-0.000*** (0.000)
Minute 45	0.474*** (0.045)	0.475*** (0.045)
Minute 90	0.933*** (0.038)	0.934*** (0.038)
Goal difference	-0.022*** (0.006)	-0.022*** (0.006)
Goal difference squared	-0.010*** (0.003)	-0.011*** (0.003)
Yellow card three minutes prior–home	-0.028 (0.031)	-0.028 (0.031)
Yellow card three minutes prior–away	0.023 (0.026)	0.023 (0.026)
Number of prior yellow cards–home	-0.017** (0.008)	-0.019** (0.008)
Number of prior yellow cards–away	0.024*** (0.008)	0.023*** (0.008)
Prior red card–home	0.045 (0.037)	0.040 (0.037)
Prior red card–away	0.024 (0.035)	0.019 (0.034)
Intercept	-2.376*** (0.098)	-2.483*** (0.102)
**Dependent variable: Yellow card**–**away**		
Additional assistant referees × Home big five		-0.035 (0.027)
Additional assistant referees × Away big five		-0.001 (0.027)
Additional assistant referees		0.060** (0.025)
Home big five	0.025* (0.014)	0.053** (0.024)
Away big five	-0.008 (0.013)	-0.003 (0.023)
Track	-0.007 (0.016)	-0.005 (0.016)
Log attendance	0.025*** (0.010)	0.028*** (0.010)
Relative strength	0.002 (0.001)	0.002 (0.001)
Relative strength squared	0.000 (0.000)	-0.000 (0.000)
Derby	0.041 (0.050)	0.042 (0.049)
Minute	0.020*** (0.001)	0.020*** (0.001)
Minute squared	-0.000*** (0.000)	-0.000*** (0.000)
Minute 45	0.493*** (0.042)	0.493*** (0.042)
Minute 90	0.962*** (0.036)	0.962*** (0.036)
Goal difference	0.007 (0.006)	0.007 (0.006)
Goal difference squared	-0.018*** (0.003)	-0.018*** (0.003)
Yellow card three minutes prior–home	0.098*** (0.025)	0.098*** (0.025)
Yellow card three minutes prior–away	-0.025 (0.026)	-0.025 (0.026)
Number of prior yellow cards–home	0.024*** (0.008)	0.023*** (0.008)
Number of prior yellow cards–away	-0.040*** (0.007)	-0.041*** (0.007)
Prior red card–home	0.100*** (0.037)	0.096*** (0.037)
Prior red card–away	0.051 (0.035)	0.047 (0.035)
Intercept	-2.809*** (0.098)	-2.885*** (0.103)
Log pseudo-likelihood	-39,746.522	-39,731.298
N	195,120	

The presented statistics are bivariate probit model estimates and standard errors, clustered at the game level, in parentheses. A definition of the variables used can be found in Section III.1.

*** (**) ((*)) indicates significance at the 1% (5%) ((10%)) significance level.

Model (1) of Table 3 is an extended version of Model (1) in Table 2. That is, indicators for home and away teams from the big five competitions are added in both blocks of the regression framework. As in [10], the likelihood of a yellow card being awarded to the home team in a given minute is higher if the away team is from the big five competitions (*b = 0*.*031*; *p = 0*.*031*). Consistently, the likelihood of a yellow card being given to the away team is (weakly significantly) higher if the home team is from the big five competitions (*b = 0*.*025*; *p = 0*.*069*).

We now turn to Model (2), which also includes, compared with Model (1), the variable capturing the presence of a fifth and sixth referee as well as interaction variables between the latter variable and the indicators of home and away teams from the big five competitions. As in our benchmark analysis, we find that the overall home favouritism can be decomposed into a statistically significant bias in games without additional assistant referees and an insignificant bias when such additional referees are added. On the one hand, when there are no additional referees, the coefficients of away teams from the big five competitions with respect to yellow cards for the home team (*b = 0*.*070*; *p = 0*.*010*) and the coefficients of home teams from the big five competitions with respect to yellow cards for the away team (*b = 0*.*028*; *p = 0*.*026*) are more substantial than the corresponding coefficients based on all games in Model (1). On the other hand, in games with additional referees, away teams from the big five competitions are no longer associated with more yellow cards for the home team (*b = 0*.*023 = 0*.*070–0*.*047*; *p = 0*.*161*), and home teams from the big five competitions are no longer associated with more yellow cards for the away team (*b = 0*.*018 = 0*.*053–0*.*035*; *p = 0*.*260*). Moreover, the introduction of additional assistant referees is again associated with additional yellow cards for both home teams (*b = 0*.*081*; *p = 0*.*004*) and away teams (*b = 0*.*060*; *p = 0*.*016*).

### IV.3 Robustness checks

To examine the robustness of these results, we conducted numerous additional analyses. First, we re-estimated Model (1) in Tables 2 and 3 for the subsample of games with and without additional assistant referees separately. Consistent with the analyses in the previous subsections, we find significant evidence for referee bias in favour of home and “big” teams only for the games without a fifth and sixth referee.

Second, we checked whether the analyses in Model (2) of Tables 2 and 3 were not just picking up a general evolution towards less referee bias over time. Therefore, we added a continuous indicator of the season both as such and in interaction with the track variable (or with the home and away team from the big five competitions variables). However, these additional variables turned out to be insignificant.

Third, as mentioned in Section III.1, the introduction of the additional assistant referees in the UEFA Europa League coincided with a new format of the group stage in this league; we are not aware of any (other) changes in UEFA instructions to referees at that time. Therefore, we re-estimated our analyses using only the games from the UEFA Champions League. In addition, we re-estimated our regression models after excluding (i) games without competitive value and (ii) return matches in the knock-out stage (in which additional time may be added). These analyses using a restricted dataset led to similar conclusions to those described in the previous subsections.

Fourth, we re-estimated our models with team and referee fixed effects. Unlike [8, 9], we did not incorporate these fixed effects into our main analyses because they result in less efficient estimates. In particular, coefficients for home and away teams from the big five competitions cannot be identified when one controls for team fixed effects. In addition, these team fixed effects remove most of the variation in our track variable. Moreover, in general, combining nonlinear models with fixed effects may yield a substantial incidental parameter problem [46, 47]. After including these fixed effects, standard errors are indeed somewhat higher compared with those in Tables 2 and 3—this is marginally the case when only referee fixed effects are added. However, coefficient estimates are highly comparable, so our conclusions remain valid when opting for fixed effects regression models.

## V. Interpretation

We find evidence for referee bias in favour of home and “big” teams when no additional referees are present. The fact that we do not find evidence of such a bias at the level of the complete dataset is driven by the fact that in our database, 70% of the games are played with additional referees. The influence of the track is heterogeneous when we differentiate between games with or without additional assistant referees. On the one hand, the presence of a track has the effect of increasing (reducing) the awarding of yellow cards to the home (away) team when no additional referees are present. This can happen if crowds that are more distant can exercise social pressure less effectively (e.g. [16]). On the other hand, after the introduction of additional referees, the tracks are less adverse (beneficial) for home (away) teams. Similar results are found in the analysis on favouritism for the teams from the big five competitions. The fact that we find an increase in yellow cards for all teams after the introduction of additional referees might be explained by a more complete observation of violations on the pitch. The better control of the game might be the reason of a lower referee bias as it may reduce the number of “uncertain” calls, which are likely the decisions with a higher risk of social sanctioning. From a broader perspective, our results are in line with [48, 49], who showed that the introduction of a second referee leads to greater enforcement of the rules in ice hockey.

We also discuss some secondary insights gained from the estimates of our control variables in the two analyses. First, if the crowd increases, the probability of the home team receiving a yellow card significantly declines, whereas the probability that the away team receives a yellow card significantly increases. Second, as the game proceeds towards the end, the probability that both teams receive a yellow card increases, albeit at a decreasing rate. Third, in minute 45 and minute 90, the probability of a sanction is significantly higher for both teams, probably as a result of the injury time that is added to these minutes. Fourth, as the goal difference increases, the probability that the home team receives a yellow card decreases. Fifth, if the home team received a yellow card in the previous three minutes, the probability of the away team receiving a yellow card increases significantly. Interestingly, if the away team received a yellow card in the previous three minutes, the chances of the home team receiving a yellow card do not increase significantly. Sixth, an extra yellow card previously received by the home team is associated with a reduction in the home team’s chances of receiving another yellow card and an increase in the away team’s chances of receiving a yellow card. Conversely, an extra yellow card previously received by the away team reduces the away team’s chances of receiving another yellow card and increases the home team’s probability of receiving a yellow card. Finally, a red card previously received by the home team is associated with a significant increase in the away team’s probability of receiving a yellow card, while a red card previously received by the away team is not associated with a significant increase in the home team’s chances of receiving a yellow card.

## VI. Conclusion

In this study, we investigated the presence of referee bias in the 2007–2014 UEFA Europa and Champions Leagues. We focused on referee bias for home teams and teams from the big five European soccer competitions, which we defined as the number of awarded yellow cards. Our estimates confirm the presence of a preferential treatment for home teams and teams from the big five competitions, which is heterogeneous on match characteristics. First, we confirmed the previous literature claiming that a running track separating the crowd from the pitch can reduce home and “big” team favouritism. Second, we investigated whether the introduction of a fifth and sixth referee in the UEFA Europa League (in the 2009–2010 season) and the UEFA Champions League (in the 2010–2011 season) reduced such a referee bias. While we found significant home and “big” team favouritism before the introduction of additional referees, no such evidence was found later. In addition, the introduction of additional assistants led to more yellow cards for both home and away teams. We showed that these findings are not just picking up a general evolution towards less bias over time and are not a result of other, parallel reforms. Finally, while our set of explanatory variables is rich, we cannot completely rule out that other unobservable information (e.g. number of fouls or penalty kicks) might affect our outcome of interest and introduce some omitted variable bias in our estimates. We acknowledge the limitation of our analysis in this respect, which is however common in the empirical literature on referee bias.

Our results suggest that the investment in two additional assistant referees by UEFA may have reduced referee bias in favour of important and home teams, which might be due to improved control over the game. However, our findings might not be generalised to investments in more referees in other contexts, let alone in better-trained or more equipped referees. Therefore, we are in favour of future work evaluating the effectiveness of other investments by UEFA or other governing bodies in this respect (such as recent investments in goal line technology or video assistant referees).

## Supporting information

S1 Dataset (XLSX)
